# Camsap3-mediated microtubules maintain transzonal projections essential for soma–germ communication during ovarian follicle maturation in mice

**DOI:** 10.1016/j.isci.2026.115911

**Published:** 2026-04-28

**Authors:** Akihiro Aikawa, Takao Tsurumaki, Erina Kuranaga, Junya Ito, Mika Toya, Masamitsu Sato

**Affiliations:** 1 Laboratory of Cytoskeletal Logistics, Department of Life Science and Medical Bioscience, Graduate School of Advanced Science and Engineering, Waseda University, Shinjuku, Tokyo, Japan; 2 Laboratory for Histogenetic Dynamics, Graduate School of Pharmaceutical Sciences, Kyoto University, Sakyo-ku, Kyoto, Japan; 3 Laboratory of Animal Reproduction, School of Veterinary Medicine, Azabu University, Sagamihara Kanagawa, Japan; 4 Graduate School of Veterinary Science, Azabu University, Sagamihara, Kanagawa, Japan; 5 Institute for Medical-Oriented Structural Biology, Waseda University, Shinjuku, Tokyo, Japan

**Keywords:** reproductive medicine, cell biology

## Abstract

Oocytes are surrounded by layers of maternal somatic granulosa cells (GCs) in ovarian follicles. GCs extend actin-containing transzonal projections (TZPs) to oocytes across the zona pellucida to establish communication. Microtubules have rarely been observed in TZPs, and their significance in TZP organization and follicular maturation remains unknown. Here, using super-resolution microscopy, we visualized microtubules alongside F-actin in most TZPs. Knockout (KO) mice of the microtubule minus-end binding protein Camsap3 (calmodulin-regulated spectrin-associated protein 3) exhibited infertility without ovulation despite normal estrous cycles. Ovaries of *Camsap3*-KO mice contained fewer developing follicles, particularly of antral and Graafian stages. In earlier stages of *Camsap3*-KO follicles, TZP numbers were reduced compared to wild-type follicles, and microtubules in TZPs were disorganized, leading to decreased contact between GCs and oocytes. TZP morphology in wild-type transforms during follicle development, and Camsap3-mediated microtubules govern the number and morphology of TZPs, contributing to successful follicle development for fertile oocyte production.

## Highlights

• Most TZPs contain microtubules at their base, with some extending along the F-actin
• TZP morphology changes during follicle development
• Camsap3-mediated microtubules maintain the TZP number and architecture
• Camsap3 contributes to the production of fertile oocytes through follicle development

## Graphical abstract

**Figure:**
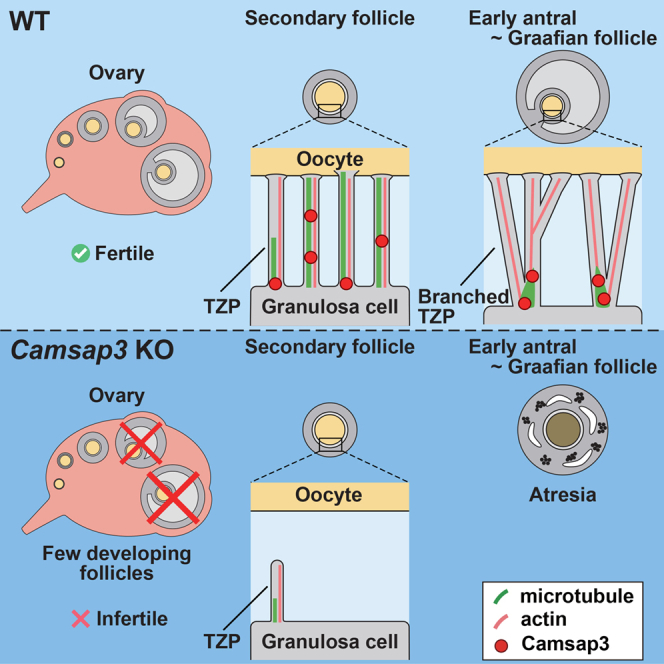

## Introduction

The oocyte is surrounded by a large number of granulosa cells (GCs) that play critical roles in the promotion of oocyte growth. The surface of the oocyte is covered by the zona pellucida, the direct contact of GCs to the oocyte is prevented. GCs extend projections, called transzonal projections (TZPs), which penetrate into the barrier of the zona pellucida and make physical contact with the cytoplasm of the oocyte via adherens and gap junctions at their tips.^1^^,^^2^^,^^3^ TZPs are thought to transfer a variety of materials and signaling molecules to the oocyte, including mRNAs, pyruvate, cGMP, and even organelles such as mitochondria, thereby regulating cell cycle control and cytoplasmic maturation of the oocyte.^4^^,^^5^^,^^6^^,^^7^^,^^8^^,^^9^^,^^10^^,^^11^ TZP is a membrane protrusion formed by cellular elongation, such as a filopodium, and contains bundles of F-actin.^3^^,^^12^ TZP was originally identified as a projection that reaches the oocyte, although the ends of some TZPs remained in the zona pellucida.^13^^,^^14^ Recent advances in microscopy have illuminated that a number of TZPs do not reach the oocyte surface^12^ and that some TZPs are branched or left unattached to the oocyte surface,^15^ indicating that the length and morphology of TZPs vary depending upon the conditions.

In several species other than mice, the presence of microtubules within TZPs has been reported.^16^ In mice, however, less than 5% of total TZPs are shown to contain microtubules,^3^^,^^16^^,^^17^ and their functional significance remained poorly understood.

Calmodulin-regulated spectrin-associated protein 3 (Camsap3) is a member of the CAMSAP family of proteins that bind to microtubule minus-end and stabilize non-centrosomal microtubules.^18^^,^^19^^,^^20^^,^^21^ Studies in mice have demonstrated that Camsap3 plays diverse roles *in vivo* across multiple tissues. In epithelial cells of the small intestine and kidney, Camsap3 orients microtubules along the apico-basal axis, thereby determining intracellular organelle positioning and the cell morphology.^22^^,^^23^^,^^24^ Camsap3 also regulates ciliary structure and the coordinated beating of motile cilia in multiciliated cells.^25^^,^^26^^,^^27^ In the nervous system, Camsap3 is known to be involved in axon differentiation and ventricular development in the brain.^28^^,^^29^

A hypomorphic mutant mouse line of *Camsap3* (*Camsap3*^*tm1a/tm1a*^) exhibits infertility in both sexes, with more severe phenotypes observed in females.^25^ Similarly, our *Camsap3*^*dc/dc*^ and *Camsap3*-KO mouse lines displayed comparable reproductive defects.^22^^,^^24^ Recent studies have linked this infertility phenotype to multiple reproductive processes. In the oviduct epithelium, Camsap3 regulates the directionality of ciliary beating required for oocyte transport.^26^ In addition, in blastomeres during early development, Camsap3 accumulates at interphase bridges between daughter cells after division, where it functions as non-centrosomal microtubule-organizing center (MTOC) and contribute to E-cadherin trafficking.^30^ Together, these findings suggest that Camsap3 functions at multiple stages of reproduction, including oocyte transport and embryo development.

In addition to these findings, our study reveals that female *Camsap3*-KO mice fail to ovulate oocytes, suggesting that Camsap3 also functions even prior to oocyte release into the oviduct; e.g., during follicular development or ovulation. Our super-resolution microscopy reveals that microtubules are present within TZPs at a higher frequency than previously detected, highlighting an underestimated role for microtubules in oocyte-GC communication.

## Results

### *Camsap3*-KO female mice were infertile due to anovulation

**Figure 1:**
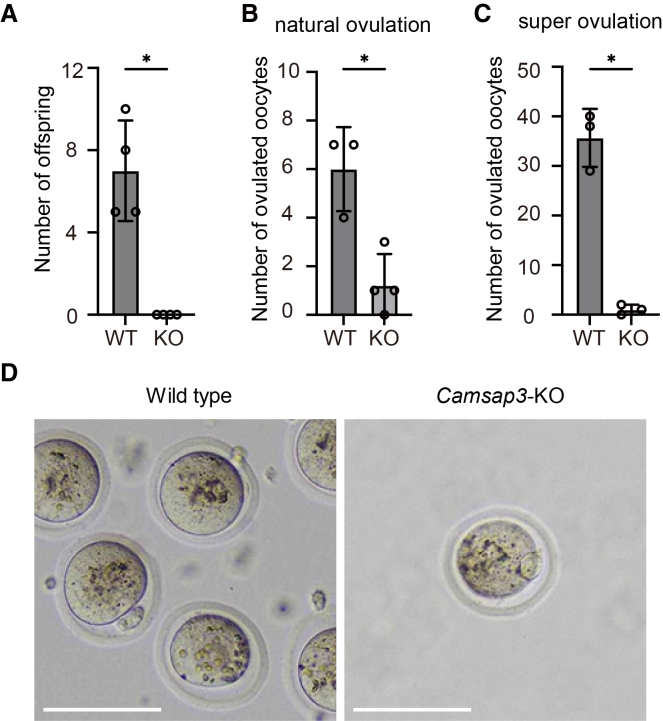
*Camsap3*-KO mice exhibit anovulation (A) The number of offspring obtained by mating WT males with WT or *Camsap3*-KO (KO) females. (WT, *n* = 4; KO, *n* = 4). WT or *Camsap3*-KO females were housed with WT males for 30 days. *Camsap3*-KO females produced no pups, although vaginal plugs were observed, as in WT females. Bars, mean; error bars, s.d. ∗*p* < 0.05, two-tailed unpaired Student’s *t* test. (B) The number of ovulated oocytes on the first day after mating (WT, *n* = 3; KO, *n* = 4). Bars, mean; error bars, s.d. ∗*p* < 0.05, two-tailed unpaired Student’s *t* test. (C) Oocyte yield after superovulation (WT, *n* = 3; KO, *n* = 3). PMSG and hCG were administered 48 h apart; oocytes were collected 12 h after hCG injection. Bars, mean; error bars, s.d. ∗*p* < 0.05, two-tailed unpaired Student’s *t* test. (D) Representative images of oocytes recovered from oviducts after superovulation. Scale bars, 100 μm. See also Figure S1.

*Camsap3* hypomorphic mutant mice *Camsap3*^*tm1a/tm1a*^ reportedly displayed subfertility in both female and male mice, with a more pronounced effect in females.^25^ Consistent with this, breeding records from the *Camsap3*-dc mutant line^22^ also indicated reduced fertility in females. To further clarify this phenotype, we employed *Camsap3*-null (*Camsap3*-KO) mice.^24^ Female *Camsap3*-KO mice were completely infertile, producing no pups despite repeated mating with wild-type (WT) males, and showed no signs of pregnancy (Figure 1A). This infertility could not be attributed to disrupted estrous cycles, as *Camsap3*-KO mice exhibited regular cycles every 4–5 days, comparable to those of WT controls.

Female mice are known to accept mating with males on the day of ovulation. To determine whether natural ovulation occurs in *Camsap3*-KO mice, we collected ovulated oocytes on the first day after mating, before implantation: 6.0 ± 1.4 oocytes were collected from WT mice, while 1.3 ± 1.1 oocytes from *Camsap3*-KO mice, suggesting that natural ovulation is impaired in the knockout (KO) mice (Figure 1B). These results suggest that infertility in *Camsap3*-KO female mice is due to a reduction in ovulated oocytes. To further assess ovulation defects, we induced superovulation with the hormones, pregnant mare serum gonadotropin (PMSG) and human chorionic gonadotropin (hCG), which promote follicle maturation and trigger ovulation. Upon superovulation, 36.7 ± 5.79 oocytes were recovered from WT mice, whereas 0.33 ± 0.47 from *Camsap3*-KO mice (Figure 1C), confirming that *Camsap3*-KO mice exhibit an anovulation phenotype, which is likely due to defects in follicle maturation in the ovaries. The few oocytes recovered from *Camsap3*-KO mice displayed morphology comparable to that of WT, with a polar body and arrest at metaphase II accompanied by a meiotic spindle (Figures 1D and S1).

### Delayed maturation after secondary follicles and increased atresia in *Camsap3*-KO mice

We examined ovarian follicle maturation in *Camsap3*-KO mice by analyzing ovarian tissue sections. We classified follicles into five stages during maturation, namely primordial, primary, secondary, early antral, and Graafian follicles, based on their morphology.^31^ After classification, we counted the number of follicles in each stage of the tissue sections from the whole ovaries.

**Figure 2:**
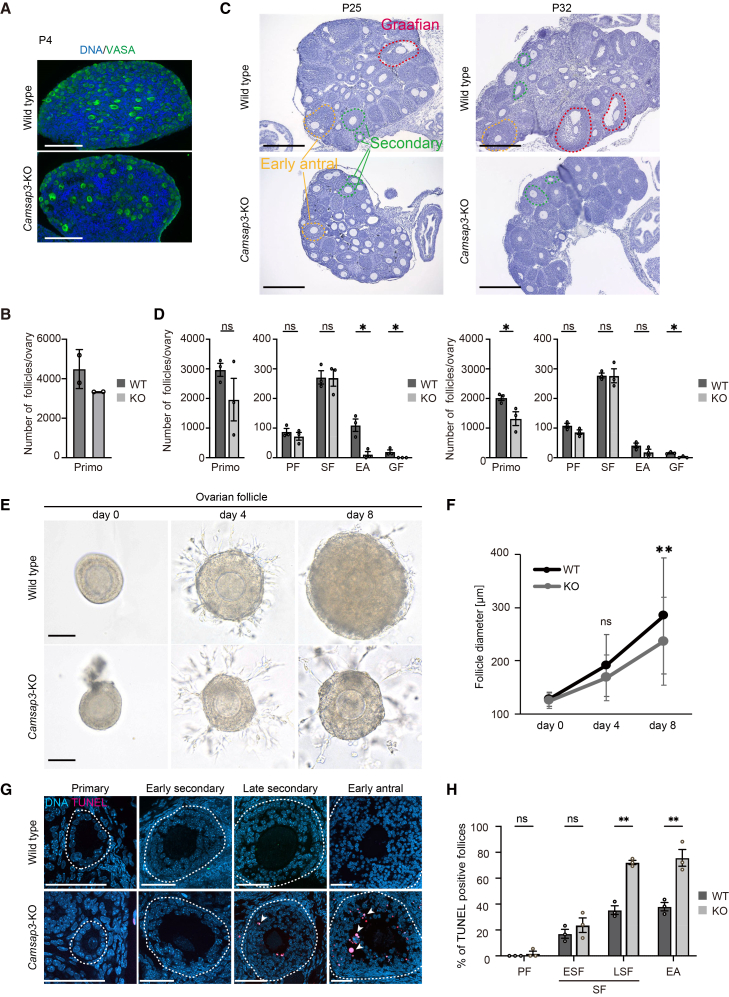
Delayed maturation after the secondary follicle stage and increased follicle atresia in *Camsap3*-KO mice (A) Ovarian sections from P4 WT and *Camsap3*-KO mice stained for VASA and DNA. Scale bars, 50 μm. (B) The number of primordial follicles per ovary (WT, *n* = 2; KO, *n* = 2). Bars, mean; error bars, s.d. (C) Ovarian sections from P25 and P32 WT and *Camsap3*-KO mice stained with hematoxylin. Scale bars, 300 μm. (D) Follicle counts at each stage: primordial (Primo), primary (PF), secondary (SF), early antral (EA), and Graafian (GF) follicles (P25, P32: WT, *n* = 3; KO, *n* = 3). Bars, mean; error bars, s.d. ∗*p* < 0.05, two-tailed unpaired Student’s *t* test. (E) Bright-field images of individual follicles encapsulated in Matrigel. Scale bars, 100 μm. (F) Average follicle diameter during Matrigel culture (WT, *n* = 39 follicles; KO, *n* = 40). Dots, mean; error bars, s.d. ∗∗*p* < 0.01, two-tailed unpaired Student’s *t* test. (G) Sections of follicles at various developmental stages stained by TUNEL. Scale bars, 50 μm. (H) Percentage of follicles undergoing regression (TUNEL-positive GCs; white arrows) at the primary, early secondary, late secondary, and early antral stages (WT, *n* = 3; KO, *n* = 3). Bars, mean; error bars, s.d. ∗∗*p* < 0.01, two-tailed unpaired Student’s *t* test. See also Figure S2.

At postnatal day (P) 4, all observed follicles were classified as primordial, with comparable numbers in WT and *Camsap3*-KO mice (4490 ± 700 in WT, 3335 ± 5 in *Camsap3*-KO; Figures 2A and 2B). Primordial follicles contain the Balbiani body (B-body), which is composed of the Golgi apparatus and is regulated by microtubules.^32^^,^^33^ B-bodies play a crucial role in regulating the activation of primordial follicles.^32^^,^^34^ As Camsap3 has been reported to localize to B-bodies,^32^ we suspected its involvement in B-body organization. However, the B-body morphology in *Camsap3*-KO was comparable to that in WT primordial follicles (Figure S2A), suggesting that there were no significant defects in primordial follicle formation.

At P25, before the first estrous cycle, when all the stages of follicles are observed in WT ovaries,^35^ the number of follicles in *Camsap3*-KO mice was comparable to that in WT mice up to the secondary follicle stage (271.3 ± 31.6 in WT, 229.6 ± 30.1 in *Camsap3*-KO; Figures 2C and 2D). However, follicles at more developed stages, the early antral and Graafian stages, were significantly reduced in *Camsap3*-KO mice (109.7 ± 29.6 in WT versus 11.3 ± 13.3 in *Camsap3*-KO in the early antral stage, and 20 ± 9.2 in WT versus 0 in *Camsap3*-KO at the Graafian stage; Figures 2C and 2D). At P32, around the time of the first estrous cycle, numbers of secondary follicles and early antral follicles in *Camsap3*-KO mice were comparable to those in WT mice (277.7 ± 11.6 in WT versus 276.3 ± 33.4 in *Camsap3*-KO at the secondary stage, and 41.7 ± 10.9 versus 18.7 ± 13.5 at the early antral stage; Figures 2C and 2D). However, Graafian follicles were significantly reduced in *Camsap3*-KO (16.0 ± 2.9 versus 2.3 ± 3.3; Figures 2C and 2D). It is possible that follicle development is delayed in *Camsap3*-KO mice, as their lighter body weight at P25 or P32^24^ potentially indicates reduced maturity compared with WT mice. To determine whether the developmental delay was due to slowed body growth rather than the loss of Camsap3 function in follicles, we examined the development of isolated secondary follicles *in vitro*. After eight days in culture, the secondary follicles isolated from WT developed to a diameter of 313.4 ± 115.3 μm, whereas those from *Camsap3*-KO mice showed limited growth to a diameter of 249.7 ± 82.7 μm, confirming that the developmental delay is due to the lack of the Camsap3 functions in follicles during the transition from secondary follicle to the early antral stage, rather than immature body conditions (Figures 2E and 2F).

At the age of 16 weeks, the period during which pregnancy is possible in mice, the proportion of follicles at various developmental stages in *Camsap3*-KO mice was comparable to that in WT follicles, although fewer Graafian follicles and corpora lutea were observed in *Camsap3*-KO mice (Figures S2B and S2C). Notably, no ovulated oocytes were observed at this stage in *Camsap3*-KO mice, unlike in WT mice, suggesting that follicle development in *Camsap3*-KO mice is impaired before reaching the Graafian stage. In ovaries, follicles that fail to develop undergo atresia, with GCs in these follicles undergoing apoptosis, as detected by TUNEL staining.^36^^,^^37^ Therefore, we suspected that follicle regression was increased in *Camsap3*-KO mice. In P32 ovaries, the number of GCs undergoing apoptosis was significantly higher in late secondary and early antral follicles of *Camsap3*-KO mice (35.3 ± 4.8% in WT versus 72.2 ± 2.3% in *Camsap3*-KO in the late secondary stage, and 38.0 ± 4.5% versus 75.7 ± 9.1% in the early antral stage; Figures 2G and 2H). These results indicated that more follicles in *Camsap3*-KO mice regressed during development and rarely developed to the Graafian stage. Consistently, HE staining revealed an increase in follicles undergoing regression, characterized by the nuclear condensation of GCs^38^ in *Camsap3*-KO mice (Figures S2D and S2E). The proportion of regressing follicles was significantly higher in *Camsap3*-KO mice than in WT mice, with 73.7 ± 12.6% versus 39.7 ± 1.5% in the late secondary stage, and 86.5 ± 9.7% versus 30.1 ± 15.1% in the early antral stage (Figures S2D and S2E). These results imply that the loss of Camsap3 decreases the developmental capability of GCs in the growing ovarian follicles.

As Camsap3 is associated with microtubules, it is possible that its KO causes spindle defects in mitotic GCs, which may result in the follicle regression. When ovarian follicles at secondary and early antral stages were immunostained for Ki67, the number of Ki67-positive GCs was comparable between WT and KO follicles at both stages (Figures S2F and S2G), indicating that GC proliferation was not detectably impaired in KO follicles. In line with this, CAMSAP3 reportedly does not localize to spindle microtubules during mitosis.^20^^,^^39^ Therefore, the follicle regression in *Camsap3*-KO is likely due to other reasons.

### Camsap3 deficiency reduces transzonal projections between GCs and the oocyte

In ovarian follicles, layers of GCs surround an oocyte and extend cytoplasmic projections known as TZPs, which penetrate the zona pellucida and reach the surface of the oocyte to establish direct contact between the GCs and the oocyte.^1^^,^^2^^,^^3^ TZPs have been reported to play a crucial role in supporting follicle maturation by facilitating intracellular communication and the transfer of essential molecules, including mRNAs and intracellular organelles such as mitochondria, from GCs to the oocyte.^5^^,^^6^^,^^7^^,^^8^^,^^9^^,^^10^^,^^11^^,^^40^ TZPs are reportedly actin-rich filopodia-like structures, less than 5% of which also contain microtubules.^3^^,^^12^ The function of microtubule-containing TZPs (tubulin-TZPs) and the organization of microtubules within them remain poorly understood.

**Figure 3:**
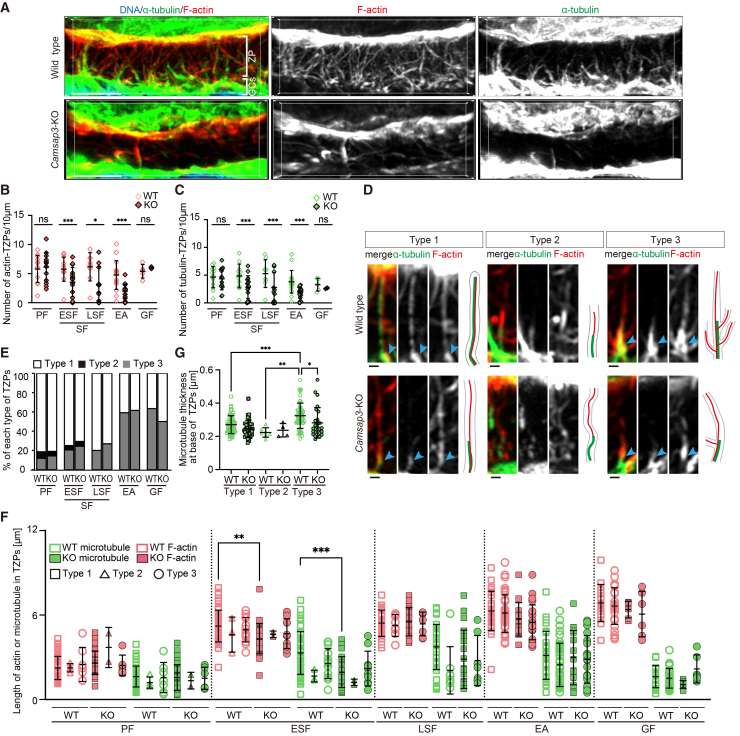
Most transzonal projections (TZPs) in WT follicles contain microtubules, and *Camsap3* deficiency reduces in the TZP number (A) Immunofluorescence images in the three-dimensional view, reconstructed from z stack confocal sections of GCs and the oocyte at the early secondary stage, stained for α-tubulin, F-actin, and DNA. Scale bars, 5 μm. (B and C) The average number of actin-TZPs and tubulin-TZPs per 10 μm at each follicle stage. The number of TZPs indicates the number of TZP roots formed from the apical surface of the cell. Numbers of follicles examined: WT, *n* = 13; KO, *n* = 13 (PF); WT, *n* = 13; KO, *n* = 15 (ESF); WT, *n* = 10; KO, *n* = 11 (LSF); WT, *n* = 17; KO, *n* = 16 (EA); WT, *n* = 5; KO, *n* = 3 (GF). Data are represented as mean ± s.d. ∗*p* < 0.05 and ∗∗∗*p* < 0.001, two-tailed unpaired Student’s *t* test. As shown in Figure 2D, in *Camsap3*-KO mice at the early antral stage, Graafian follicles were rare “survivors,” and TZPs were assessed in these remaining follicles. (D) Representative images of three types of microtubule-actin arrangements. Arrowheads indicate microtubule bases from which F-actin branches extend. Scale bars, 0.5 μm. (E) The percentage of each TZP type (as defined in Figure 3D) by developmental stage. (F) F-actin and microtubule lengths of each TZP type at five stages of follicle development. Numbers of GCs examined: WT, *n* = 10; KO, *n* = 10 (PF); WT, *n* = 10; KO, *n* = 10 (ESF); WT, *n* = 10; KO, *n* = 10 (LSF); WT, *n* = 10; KO, *n* = 10 (EA); WT, *n* = 10; KO, *n* = 3 (GF). Statistical analysis was performed using multi-group comparison of F-actin and microtubule datasets for each developmental stage. Data are represented as mean ± s.d. ∗∗*p* < 0.01 and ∗∗∗*p* < 0.001, one-way ANOVA. (G) Average microtubule thickness at the base of TZPs. Numbers of TZPs examined: WT, *n* = 94; KO, *n* = 80 (type 1); WT, *n* = 7; KO, *n* = 4 (type 2); WT, *n* = 73; KO, *n* = 45 (type 3). Data are represented as mean ± s.d. ∗*p* < 0.05, ∗∗*p* < 0.01 and ∗∗∗*p* < 0.001, two-tailed unpaired Student’s *t* test. See also Figure S3.

In *Camsap3*-KO follicles, the number of TZPs detected by F-actin (actin-TZPs) was initially similar to that in WT follicles at the primary stage (5.8 ± 2.2 in WT vs. 6.1 ± 2.4 in *Camsap3*-KO), and then significantly reduced at the early secondary stage, which is right before the number of follicles begins to decline in *Camsap3*-KO (Figures 3A and 3B). This tendency lasted through the early secondary stage (5.8 ± 2.0 vs. 3.5 ± 2.4) until the early antral stage (4.8 ± 2.4 vs. 2.0 ± 1.2; Figures 3B and S3A–S3D). In Graafian follicles, the number of actin-TZPs in *Camsap3*-KO was comparable to that in WT (Figure 3B). Given that the number of follicles per se declined in *Camsap3*-KO at the early antral stage, Graafian follicles counted here were rare “survivors” (Figures 2C and 2D). As the number of actin-TZPs in those survivors was comparable to that in WT Graafian follicles, only follicles retaining actin-TZPs might be able to reach the Graafian stage. The decrease and abnormalities of TZPs in *Camsap3*-KO were found even at the early secondary follicle stage (Figures 3A and 3B), whereas an increase of GC apoptosis was detected from the late secondary follicle stage onwards (Figures 2G and 2H). Thus, the temporal sequence indicates the possibility that TZP abnormalities precede the increase in GC apoptosis, although the causality remains to be elucidated.

These observations suggest that Camsap3-mediated “microtubules” play a more crucial role in maintaining TZPs than was previously understood.

### Super-resolution inspection illuminates the co-existence of microtubules and actin in most TZPs

In line with the significance indicated above, our super-resolution microscopy revealed that more than 80% of TZPs in WT follicles contained microtubules, in addition to F-actin (Figures 3A–3C), although microtubules appeared relatively shorter than F-actin in TZPs (see later in discussion for details). In WT follicles, the density of tubulin-TZPs peaked in the late secondary stage and then gradually decreased as follicle development progressed (Figure 3C).

In *Camsap3*-KO, the density of tubulin-TZPs was initially comparable to that in WT at the primary stage, but was lower than in WT from the early secondary to the early antral stages ––– the numbers of tubulin-TZPs per 10 μm of the luminal surface were: 4.6 ± 1.8 in WT vs. 4.3 ± 1.9 in *Camsap3*-KO at the primary stage; 4.9 ± 2.0 vs. 2.8 ± 2.0 at the early secondary stage; 5.3 ± 2.6 vs. 2.8 ± 2.5 in late secondary stage; 3.8 ± 2.0 vs. 1.8 ± 1.0 at the early antral stage (Figure 3C). This suggests that Camsap3-mediated microtubules may play a role in TZPs in the secondary follicle stage, specifically or later. As the timing precedes the significant reduction of *Camsap3*-KO follicles in ovaries, defects in tubulin-TZPs caused by a loss of Camsap3 in secondary follicles impact subsequent follicle maturation in *Camsap3*-KO.

We characterized three distinct patterns regarding the internal architecture of TZPs that containing microtubules: type 1) microtubules and F-actin run alongside for most of the TZP length; type 2) microtubules are localized at the basal portion of the TZP, from the top of which F-actin extends toward the TZP tip; and type 3) F-actin branches off from the middle of a thick microtubule base such as a “tree-trunk” (Figure 3D). In both WT and *Camsap3*-KO follicles, all three types of TZPs were observed in the early stages of follicle development, with type 1 being the most dominant (over 70% of all tubulin-TZPs; Figure 3E). Type 2-TZPs diminished as follicle development progressed and disappeared by the late secondary stage, while the ratio of type 3-TZPs gradually increased up to over 60% at the Graafian stage (Figure 3E). The temporal transition of the ratios of the three TZP patterns during follicle development was equivalent in WT and *Camsap3*-KO follicles (Figure 3E).

The length of F-actin in TZPs increased as WT follicles grew (magenta, WT; Figure 3F), whereas the length of the microtubules decreased after the late secondary stage (green, WT), demonstrating that F-actin and microtubules were temporally rearranged according to stages to possibly fulfill specific functions for follicle maturation at each stage. In *Camsap3*-KO, microtubules, particularly those of type 1-TZPs, remained short in the early secondary follicles (green squares, Figure 3F). In WT follicles, microtubules at the bottom of type 3-TZPs were thicker than those in other types, whereas those in *Camsap3*-KO follicles were thin, similar to those in the other two types (Figure 3G). Thus, Camsap3 appears to play a role in the maintenance of microtubule length, particularly in type 1-TZPs during the early secondary stage, and in the thickness of microtubules in type 3-TZPs.

In type 2- and 3-TZPs, microtubules extend from GCs, with their distal ends closely associated with the proximal ends of the actin filaments. The actin filaments then extend from the tip or lateral surface of the microtubules toward the oocyte. A scheme including the microtubule-plus-end-binding protein CLIP-170 and the actin-nucleator formin may operate at the sites for the microtubule-actin interplay by analogy: CLIP-170 reportedly interacts with mDia1, a member of the formin family, and recruits it to the microtubule plus-end so that it accelerates actin polymerization therefrom.^41^^,^^42^ Under these conditions, CLIP-170 and mDia1 tracked the growing ends of the actin filaments.^42^ Indeed, in the WT,13% of type 1-, 4.0% of type 2-, and 13% of type 3-TZPs displayed a CLIP-170 signal at the tip or lateral surface of actin filaments (Figures S3E–S3G), suggesting that microtubules in TZPs contribute to the nucleation and growth of actin filaments, allowing them to reach the oocyte more efficiently than TZPs consisting of microtubules only. Although CLIP-170 was detected in a limited manner at the microtubule-F-actin interface, this was due to transient localization, as previously reported *in vitro.*^42^

Mural granulosa cells (mGCs) were found to possess randomly oriented cytoplasmic projections that are similar to TZPs,^12^^,^^43^ important for ovarian follicle development. We therefore examined whether projections of mCGs that did not contact neighboring GCs are affected by *Camsap3*-KO in antral follicles. Staining for F-actin and microtubules revealed that the number of cytoplasmic projections in mGCs was comparable between WT and *Camsap3*-KO follicles (10 ± 1.4 in WT vs. 9.22 ± 1.3 in *Camsap3*-KO, quantified based on F-actin staining; Figures S3H and S3I). Notably, approximately 38% of the mGC projections contained detectable microtubule signals in WT, which is substantially lower than in TZPs, where more than 80% contain microtubules. These observations suggest that Camsap3-mediated microtubule stabilization may play a more prominent role in structural maintenance of TZPs than in assembly of cytoplasmic projections of mGCs.

Oocyte-secreted factors (OSFs), such as growth differentiation factor 9 (GDF9), regulate the formation of TZPs.^3^^,^^44^ Oocyte-derived microvilli (Oo-Mvi) enrich and release OSFs to stimulate GCs.^45^ In *Camsap3*-KO follicles, Oo-Mvi visualized with radixin staining were indistinguishable from those in WT, and GDF9 levels in the oocyte were comparable to WT in immunofluorescence (Figures S3J–S3M). These findings suggest that the lack of OSFs is unlikely to account for the reduction in TZPs observed in *Camsap3*-KO follicles. Rather, Camsap3 appears to contribute to the organization and maintenance of TZP structure.

To further focus on the contribution of the oocyte and GCs for follicle development, we performed *in vitro* reconstitution of chimeric follicles with a combination of oocytes and GCs derived from WT and *Camsap3*-KO mice (Figure S3N). When follicles were reconstituted using KO GCs and a WT oocyte, a significant reduction of the follicle size was observed (GCs: KO + Oocyte; WT, Figures S3N–S3P). The counter-combination of WT GCs and a KO oocyte also tended to reduce the follicle size, although not statistically significant. These results indicate that Camsap3 in GCs makes a contribution to follicular development, and that Camsap3 in oocytes may independently contribute to follicular development, although the mechanism remains to be elucidated.

### TZPs contain Camsap3-associated microtubules with mixed polarity that maintain GC-oocyte connection

**Figure 4:**
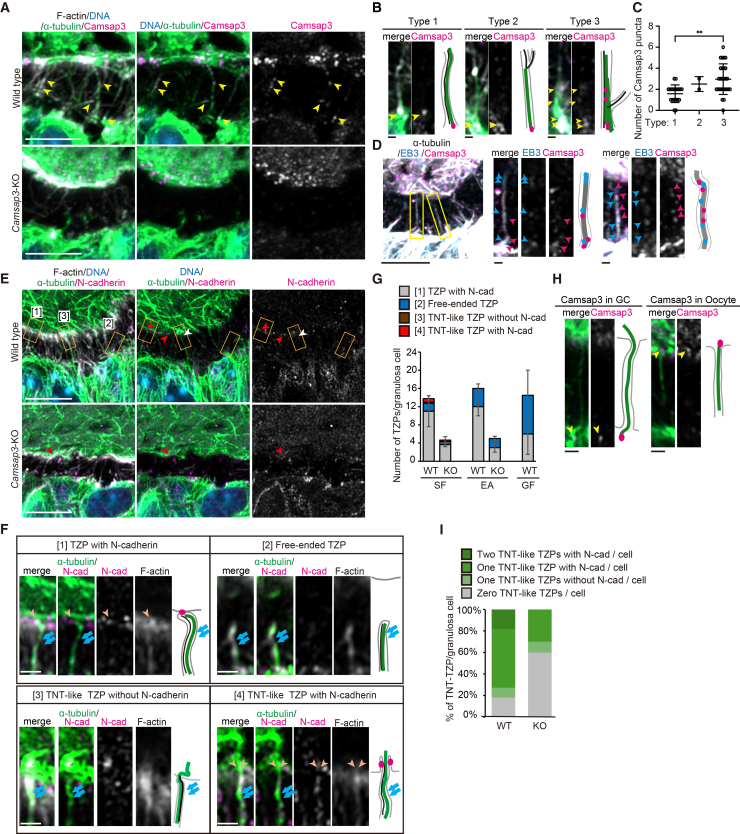
TZPs contain Camsap3-associated microtubules of mixed polarity that maintain oocyte-GC connections (A) Immunofluorescence images of GCs and an oocyte at the early secondary follicle stage, stained for α-tubulin and Camsap3 (arrowheads). Immunostaining with the antibody for Camsap3 reportedly shows non-specific signals as detected in *Camsap3*-KO,^19^ and Camsap3 localization is based on staining that is associated with microtubules within TZPs in WT follicles, a pattern that is absent in KO follicles. Scale bars, 5 μm. (B) Representative images of the three types of TZPs. Camsap3 (arrowheads) localized to the base of the TZP and along the TZP. Scale bars, 0.5 μm. (C) The number of Camsap3 puncta localized per TZP for each type (type 1, *n* = 19 TZPs; type 2, *n* = 2, type 3, *n* = 27). Data are represented as mean ± s.d. ∗∗*p* < 0.01, two-tailed unpaired Student’s *t* test. (D) Immunostaining for Camsap3 (magenta arrowheads), EB3 (blue arrowheads), and α-tubulin revealed that microtubules within TZPs displayed both plus and minus ends on the oocyte side. Insets are magnified and shown on the right. Scale bars, 5 μm (left) and 0.5 μm (the others). (E) Representative images of N-cadherin foci on the oocyte surface at the early secondary follicle stage. Insets [1] – [3] indicate representative TZPs which belong to type [1] – [3], respectively. Arrowheads indicate the position of TZP ends on the surface of the oocyte with (red) or without (white) N-cadherin signals. Scale bars, 5 μm. (F) Representative images of the contact area between an oocyte and TZPs: [1] N-cadherin localized to TZP tips in contact with the oocyte (arrowheads); [2] free-ended TZPs not connected to the oocyte; [3] TNT-like TZPs with microtubules penetrating the oocyte without N-cadherin; [4] TNT-like TZPs with microtubules penetrating the oocyte accompanied by N-cadherin (arrowheads). Arrows indicate microtubules inside TZPs. Scale bars, 0.5 μm. (G) Quantification of TZP types is shown in (F) at each developmental stage. Error bars, s.d. (H) Representative images of Camsap3 localization (arrowheads) to TNT-like TZPs. Camsap3 localized to the TZP base inside the GC or to the TZP tip inside the oocyte. Scale bars, 0.5 μm. (I) Proportion of TNT-like TZPs per GC. See also Figure S4.

Immunofluorescent staining showed that, in WT follicles, Camsap3 localized to TZPs and the apical region of GCs at all stages of follicle development (Figures 4A and S4A–S4E). Camsap3 signals were also observed on the surface of the oocyte, although some appeared non-specific (Figure 4A). Camsap3 is located at the base of microtubules near the apical surface of GCs, along the microtubules, and at the ends of microtubules toward the oocyte in all three types of TZPs (Figure 4B). Type 3-TZPs contained a significantly higher number of Camsap3 puncta at the bottom of the thick microtubule “tree-trunks” than type 1- and type 2-TZPs (Figures 4B and 4C). Double-staining of Camsap3, which associates with the minus-end of non-centrosomal microtubules,^18^^,^^19^^,^^20^ and the plus-end marker EB3 revealed that each TZP contained several microtubules of mixed polarity (Figure 4D). Magnified three-dimensional views from the apical side of GCs revealed that microtubules extended from the vicinity of intercellular junctions as well as from the middle of the apical surface in WT follicles (Figure S4F). In contrast, in *Camsap3*-KO GCs, TZPs tended to extend from the middle if present (Figure S4F). The remaining TZPs contained microtubules mediated by either the centrosome or Camsap2 but not Camsap1 (Figures S4G–S4J), suggesting that Camsap3, in cooperation with Camsap2 and the centrosome, stabilizes minus-ends of the microtubules to maintain their organization. The localization of Camsap2 (and Camsap1) in *Camsap3*-KO ovaries was not due to compensatory protein upregulation, as protein levels of Camsap1 and Camsap2 in *Camsap3*-KO were indistinguishable from those in WT (Figure S4K). Therefore, the observed phenotypes regarding TZPs in *Camsap3*-KO ovaries reflect a specific requirement for Camsap3.

At the TZP-oocyte interface, TZPs extended from GCs present N-cadherin, whereas the oocyte presents E-cadherin,^46^ along with gap junctions displayed in both cell types.^47^ The gap junction protein connexin Cx37 presented on the surface of WT oocytes,^48^ was reduced on that of KO (Figures S4L and S4M). Reflecting them, most TZPs of the secondary stage terminated at the oocyte surface, and some even penetrated the interior (Figures 4E and 4F), as previously described for Graafian follicles.^49^ In the secondary stage when type 1-TZPs dominated, 74.1% of TZPs terminated at the oocyte surface, as indicated by the presence of N-cadherin ([1], Figures 4E–4G). In contrast, 7.3% of TZPs showed projection of the microtubule signal into the oocyte, with ([4]) or without ([3]) N-cadherin signals, suggesting that these TZPs may represent open-ended tunneling nanotube (TNT)-like projections. TNT is a thin membranous structure that provides direct cytoplasmic connections between distant cells, enabling direct transfer of organelles and vesicles.^50^ TNT-like projections displayed Camsap3 at the bottom end of penetrating microtubules (Figure 4H). In addition, Camsap3 was occasionally observed at the tip of TNT-like TZPs facing the oocyte, reflecting the mixed polarity of randomly oriented internal microtubules. As TNT-like TZPs continuously connect the cytoplasm of GCs and the oocyte, it is possible that tubulin and microtubules derived from the oocyte partly contribute to the assembly of microtubules detected within TZPs, which may account for the extension of microtubules with mixed polarity into the oocyte.

When the number of microtubules remained small in *Camsap3*-KO, GCs without any TNT-like projections substantially increased (60.0% in KO vs. 18.2% in WT; Figure 4I). Cell organelles such as mitochondria and mRNAs have been reported to translocate through TZPs.^8^^,^^9^^,^^10^^,^^11^^,^^40^ We speculate that TNT-like TZPs may be selectively used for the translocation of these macromolecules because they cannot pass through cadherin-connected junctions. Thus, the reduction of TZPs in *Camsap3*-KO, particularly the substantial loss of TNT-like projections, may cause severe deficiency in macromolecule translocation, which affects follicle maturation at an early stage.

## Discussion

This work demonstrates that Camsap3 contributes to follicle maturation through the organization of TZPs, including TNT-like projections at the early stage of maturation, which is essential for GC-oocyte (soma-germline) communication. This explains the infertility phenotype caused by Camsap3 dysfunction in the female mice. *Camsap3*-KO follicles exhibited a reduced number of TZPs, and our super-resolution microscopy with optimized fixation for microtubules revealed that most TZPs contain microtubules. This defies the conventional view of TZP as actin-dominated specialized filopodia, since the existence of microtubules was underestimated to be only 5%. We thus unmasked the hidden significance of microtubules in TZP organization. *Camsap3*-KO follicles in the secondary stage showed a substantial decrease in TNT-like TZPs, accompanied by an increase in follicle atresia, suggesting that inefficient communication between GCs and the oocyte via tubulin-TZPs may primarily cause the symptoms, although additional contributions from other factors cannot be excluded. Although most *Camsap3*-KO follicles failed to reach the Graafian stage with a substantial loss of TNT-like TZPs, a few normal follicles with TZPs were found, in which Camsap2 may have compensated for the loss of Camsap3 function. Nonetheless, almost no ovulated oocytes were collected from *Camsap3*-KO mice, indicating that Camsap3 plays a crucial role in follicle maturation.

TZP contacts the oocyte surface via gap junctions and adherence junctions, through which molecules required for oocyte growth, such as pyruvate or cGMP, are transferred from GCs to oocytes.^17^^,^^51^ A previous study detected mitochondria within TZPs in transmission electron microscopy (TEM).^10^ Although it remains unclear how larger biomacromolecules, including mitochondria, are transported through cellular junctions, microtubule-prominent TZPs may be key to their transport. TZPs that form end-on contacts with the oocyte may transport mitochondria into the oocyte via vesicular trafficking mechanisms, whereas TZPs that penetrate the oocyte may deliver them directly into the oocyte cytoplasm, implying that TNT-like TZPs, rather than end-on TZPs, might be the primary pathway for mitochondrial transport. Tom20 localization representing mitochondria was observed in TZPs containing microtubules, particularly in type 1-TZPs that include TNT-like ones, but not in TZPs with actin filaments only, supporting this hypothesis (Figures S4N and S4O). TNT-like tubulin-TZPs are observed only in the early stage of follicle development, suggesting that larger components essential for oocyte growth are efficiently transferred at this stage via microtubules that potentially facilitate directional transport.

This study demonstrated the temporal transition of TZPs during follicular development. In early stages, the majority of TZPs were straight and contained both microtubules and F-actin (Figure S4P), which may be suitable for macromolecular transport into oocytes. In contrast, in the later stages, branched TZPs with shorter microtubules increased. Branched TZPs reaching the oocyte surface may increase the area of physical contact through which relatively smaller molecules may be efficiently transferred, thereby controlling GCs to prepare for ovulation. The prevalence of thin and branched TZPs in the late stages may also facilitate timely degradation prior to ovulation. F-actin is the major component in the branches, but nonetheless, “tree-trunk” microtubules serve as the platform for the growth of the F-actin branches. Although TZPs thus transform over time, it is remarkable that Camsap3-mediated microtubules play a pivotal role in the assembly of both types of TZPs.

### Limitations of the study

This study reveals that more TZPs include microtubules than previously considered by the use of super-resolution microscopy. The microtubule minus-end protein Camsap3 localizes to microtubules inside TZPs, and the *Camsap3*-KO ovary is deficient in the formation and morphology of TZPs. Although the defects of TZPs in *Camsap3*-KO follicles are remarkable, it remains to be elucidated how it affects the development of ovarian follicles. First, it remains unclear what kind of molecules are transported between the oocyte and GCs in WT ovarian follicles, as well as how the transport is affected by *Camsap3* knockout. Furthermore, the role of microtubules in the assembly of TZPs requires further investigation. While this study highlighted the presence of microtubules in most TZPs, they are often shorter than F-actin within these structures. It remains unclear whether the microtubules are dynamically growing or shortening, and how they contribute to the assembly of TZPs that contain both microtubules and F-actin. Our reconstitution assays indicate that Camsap3 is essential in both GCs and oocytes. Although this study concentrated on its roles in TZP organization extended from GCs, it is equally important to explore the functions of Camsap3 in oocytes for follicle maturation. Once we have gained the insight, we will be able to demonstrate the direct causality between the TZP defects in *Camsap3*-KO female mice and their infertility, which we have not yet clarified with direct evidence at this stage.

## Resource availability

### Lead contact

Further information and requests for resources should be directed to and will be fulfilled by the lead contact: Masamitsu Sato (masasato@waseda.jp).

### Materials availability

This study did not generate new unique reagents, and all materials in this study are commercially available.

Any additional information required to reanalyze the data reported in this paper is available from the lead contact upon request.

### Data and code availability

• All data reported in this paper will be shared by the lead contact upon request.
• This paper does not report original code.
• Any additional information required to reanalyze the data reported in this paper is available from the lead contact upon request.

## Acknowledgments

We thank Masatoshi Takeichi (RIKEN BDR) for experimental materials, valuable discussions, and helpful suggestions. We are also grateful to Kanako Tsuzuki and Sonoko Saji for their contribution to the initial stages of this study. A.A. was supported by JST SPRING, grant no. B2R101263201. This work was supported by 10.13039/501100001691JSPS KAKENHI grant nos. 25K09625 to M.T., and 16H04787, 18K19347, 23K27173, and 25H02582 to M.S. Support also came from the Daiichi Sankyo Foundation of Life Science to M.S, Ohsumi Frontier Science Foundation to M.T., and Waseda University grants for Special Research Projects grant nos. 2022C-170, 2023Q-012, 2024R-029 (to M.T.), 2017B-243, 2020R-038, 2023C-167, 2024C-490, 2025R-070, and 2025C-154 (to M.S.).

## Author contributions

A.A.: investigation, methodology, validation, visualization, and writing – original draft; T.T.: investigation, methodology, validation, visualization, and writing-review and editing; E.K.: project administration, resources, and supervision,; J.I.: conceptualization and project administration; M.T.: conceptualization, funding acquisition, project administration, supervision, writing – original draft, and writing-review and editing; M.S.: conceptualization, funding acquisition, project administration, supervision, writing – original draft, and writing-review and editing.

## Declaration of interests

The authors declare no competing interests.

## STAR★Methods

### Key resources table

**Table:** 

REAGENT or RESOURCE	SOURCE	IDENTIFIER
**Antibodies**		

Donkey anti-goat IgG (H+L) Highly Cross-Adsorbed Secondary Antibody, Alexa Fluor 488	Jackson Immuno Research	705-545-147; RRID: AB_2336933
Goat anti-mouse IgG (H+L) Highly Cross-Adsorbed Secondary Antibody, Alexa Fluor 555	Thermo Fisher	A32727; RRID: AB_2633276
Goat anti-mouse IgG (H+L) Highly Cross-Adsorbed Secondary Antibody, Alexa Fluor 647	Thermo Fisher	A32728; RRID: AB_2633277
Goat anti-Rabbit IgG (H+L) Highly Cross-Adsorbed Secondary Antibody, Alexa Fluor 555	Thermo Fisher	A32732; RRID: AB_2633281
Goat anti-Rabbit IgG (H+L) Highly Cross-Adsorbed Secondary Antibody, Alexa Fluor 647	Thermo Fisher	A32733; RRID: AB_2633282
Goat anti-Rat IgG (H+L) Highly Cross-Adsorbed Secondary Antibody, Alexa Fluor 647	Thermo Fisher	A48265; RRID: AB_2895299
Peroxidase AffiniPure Sheep anti-mouse IgG (H+L)	Jackson Immuno Research Laboratories	515-035-003; RRID: AB_2340295
Donkey anti-Rabbit IgG, HRP-Linked Whole Ab	cytiva	NA934; RRID: AB_772206
Rabbit anti-VASA	abcam	ab13840; RRID: AB_443012
Mouse anti-CLIP170	Sanra cruz	sc-28325; RRID: AB_671001
Mouse anti-GM130	BD Bioscience	610823; RRID: AB_398142
Mouse anti-α-tubulin-FITC	Sigma	F2168; RRID: AB_476967
Rabbit anti-CAMSAP1	Novus Bio	NBP1-26645; RRID: AB_1852845
Rabbit anti-CAMSAP2	Protein tech	17880-1-AP; RRID: AB_2068826
Rabbit anti-CAMSAP3	Tanaka et al. (ref.^19^)	
Alexa Fluor 594 Rabbit anti-TOMM20	Abcam	ab210665; RRID: N/A
Rabbit anti-GDF9	R&D	AF739; RRID: AB_2111517
Rabbit anti-N-cadherin	Abcam	ab18203; RRID: AB_444317
Rabbit anti-Radixin	Abcam	ab52495; RRID: AB_882259
Rat anti-EB3	Abcam	ab53360; RRID: AB_880026
Rabbit anti-Pericentrin	Abcam	ab4448; RRID: AB_304461
Mouse anti-GAPDH	MBL Life science	M171-3; RRID: AB_10597731
Rabbit anti-Connexin37	Thermo Fisher	40-4300; RRID: AB_2533465

**Chemicals, peptides, and recombinant proteins**		

Alexa Fluor 647 Phalloidin	Thermo Fisher	A30107
DAPI solution	Dojindo	340-07971
hCG	ASKA Pharmaceutical Co., Ltd.	28-0162
PMSG	ASKA Pharmaceutical Co., Ltd.	28-0041
α-MEM nucleosides, GlutaMax	Thermo Fisher	32571036
FSH Gonal-F	Merck	4987496300011
FBS	NICHIREI	175012
ITS (insulin-transferrin-selenium)	Sigma	I3146
Matrigel	Becton Dickinson	354234
Hyaluronidase	Sigma	H4272
Mayer's Hematoxylin Solution	FUJIFILM Wako	132-09665
VECTASHIELD	Vector laboratories	H-1000
Prolong Glass	Thermo Fisher	P36980
OCT compound	Sakura Finetek Japan	45833
SuperSep Ace	FUJIFILM Wako	197-15011
Immobilon -P PVDF Membrane	Millipore	IPVH00010
Stripping Solution	FUJIFILM Wako	193-16375

**Critical commercial assays**		

In Situ Cell Death Detection Kit, Fluorescein	Merck	11684795910

**Experimental models: Organisms/strains**		

*Camsap3*-KO mice	Mitsuhata et al.^24^	RIKEN BRC: CDB0033E

**Software and algorithms**		

Imaris	Oxford instrument	https://imaris.oxinst.com/ RRID: SCR_007370
Fiji/ImageJ	Schindelin et al. (ref.^52^)	https://fiji.sc/
Zen	Zeiss	https://www.zeiss.com/microscopy/en/products/software/zeiss-zen.html RRID:SCR_013672
BZ-X Analyzer	Keyence	https://www.keyence.co.jp/support/bio/analyzer/
GraphPad Prism	GraphPad	http://www.graphpad.com/

**Other**		

ImageQuant LAS 500	cytiva	LAS 500
BZ-X710 All-in-One Fluorescence Microscope	Keyence	BZ-X710
LSM980 laser scanning confocal microscope with Airyscan 2	Zeiss	LSM980
ECLIPSE Ti2	Nikon	ECLIPSE Ti2

### Experimental model and study participant details

#### Mice

*Camsap3*-knockout (*Camsap3*-KO) mice used in this study were generated as described in our previous study (RIKEN BRC: CDB0033E),^24^ in which the entire genomic region encoding Camsap3 was deleted. N3 and N4 generation mice were used in this study. Backcrossing was performed using C57BL/6NCrSlc mice. Wild-type (WT) and *Camsap3*-KO female mice aged 0–17 weeks were analyzed. For ovarian follicle culture, female mice aged 2–5 weeks were used, as described below. WT male mice aged 8 weeks or older were used for fertility tests. Mice were housed in a specific pathogen-free (SPF) room. 8:00-20:00 lighted. The humidity was approximately 30-70 %, and the temperature was approximately 20°C. Use of these mice was approved by the Waseda University Animal Review Committee; approval numbers A24-082, A24-083, A25-078 and A25-079. Management and experiments were conducted in accordance with the guidelines and protocols provided by the committee.

### Method details

#### Genotyping

Genotyping of *Camsap3*-KO mice was performed by PCR as described previously.^24^ Tail biopsies were used to extract template DNA. To distinguish between WT and *Camsap3*-KO alleles, three primers (P1, P2, P3) reported in Mitsuhata et al., 2021^24^ were used. PCR amplification with P1/P2 generated a 411-bp product corresponding to WT allele, whereas amplification with P1/P3 yielded a 614-bp product corresponding to the *Camsap3*-KO allele. The P1/P3 region in WT mice was not amplified under the PCR conditions used.

#### Fertility test

Female mice were tested between 8 and 17 weeks of age, and male mice were tested from 8 weeks of age onwards. For crossbreeding, males larger than females were selected. One female and one male were co-housed for one month, and females were examined daily for the presence of vaginal plugs. The mating combinations were: (1) ♂ WT × ♀ WT, (2) ♂ WT × ♀ *Camsap3*-KO. Successful copulation was confirmed by the presence of a vaginal plug, after which the pair was separated. Mice typically give birth 19 days after successful copulation.

#### Natural ovulation and superovulation

For superovulation treatment, 8-weeks-old WT and *Camsap3*-KO mice were injected with 10 IU PMSG (ASKA Pharmaceutical) at 17:00, followed 48 h later by 5 IU hCG (ASKA Pharmaceutical). Mice were dissected 12 h after hCG administration. Cumulus cells were removed using 50 mM hyaluronidase (Sigma), and the number of oocytes were counted. For natural ovulation, oocytes were collected the morning after confirmation of a vaginal plug.

#### *In vitro* growth of ovarian follicle

As previously reported,^45^ follicles were grown for maturation in a three-dimensional culture system using Matrigel (Becton Dickinson). Matrigel was mixed with culture medium (3:1) on ice; the medium consisted of FSH (10 mIU/ml; Merck), ITS (Sigma), and 10 % FBS (NICHIREI) in MEMα supplemented with GlutaMax (Thermo Fisher). Drops of the mixture (20 μl) were placed at the bottom of a 35-mm Petri dishes and incubated for 20 min to solidify. Follicles of approximately 130 μm in diameter were isolated from 2–5 week-old mice in MEMα and embedded in the gel using a mouth pipette. Cavities generated during the process were sealed with 2 μl of additional Matrigel. Each dish was filled with 1 ml of the culture medium and incubated at 37°C with 5 % CO_2_ for 8 days, with half of the medium replaced every other day.

#### Hematoxylin staining

Tissue samples were prepared as previously described,^53^ with minor modifications. Glass slides with paraffin sections were first soaked in xylene for 5 min, followed by soaked twice for 3 min each. The samples were sequentially immersed twice in 100 % ethanol for 3 min, 90 % ethanol for 3 min, 80 % ethanol for 3 min, followed by 70 % ethanol for 3 min. After immersion in hematoxylin solution (Wako) for 3 min, the sections were washed in tap water for a few seconds and then transferred to tap water at 30°C for 15 min. The samples were washed with 100 % ethanol. The samples were then soaked in ddH_2_O with manual shaking constantly for 15 min and mounted with glycerol.

#### TUNEL assay

Paraffin sections of 5 μm thickness were used. For deparaffinization, the sections on glass slides were placed on a heating plate at 55°C for 30 min, followed by two sequential 15-min immersions in xylene. To remove xylene, the samples were sequentially immersed twice in 100 % ethanol for 5 min, 90 % ethanol for 5 min, 80 % ethanol for 5 min, and 70 % ethanol for 5 min. The samples were then immersed in ddH_2_O for at least 20 min. The samples were treated with Proteinase K solution (10–20 μg/ml in 10 mM Tris/HCl, pH 8) and incubated at 37°C for 30 min, followed by wash with PBS for three times. The samples were dried up, and 50 μl of TUNEL reaction solution (Merck) was applied. The samples were incubated at 37°C for 60 min in the dark. VECTASHIELD mounting medium (Vector laboratories) was used for the encapsulation.

#### Immunofluorescence staining

Tissue samples were prepared as previously described,^22^ with minor modifications. Ovarian tissue was fixed by immersion in 2 % PFA/50 mM Sorbitol/PEM for 1 h at room temperature and then washed three times for 10 min each in PEM buffer. For cryoprotection, tissues were sequentially transferred to sucrose/PEM solutions: 15% for 2 h at 4°C, 20 % overnight at 4°C, and 30 % for 5 h at 4°C. Samples were embedded in OCT compound (Sakura Finetek Japan) and snap-frozen, in liquid nitrogen, then stored at –80°C. Frozen blocks were sectioned at 5 μm using a CM1950 (Leica).

Glass slides with frozen ovarian sections were incubated in 0.1 % Triton X-100/PEM for 10 min at room temperature, followed by permeabilization in 0.2 % Triton X-100/PEM for 10 min. Samples were then washed in 0.1 % Triton X-100/PEM with shaking for 10 min. Blocking was performed with 3 % bovine serum albumin (BSA)/0.1 % Triton X-100/PEM for 1 h at room temperature. Primary antibodies, diluted in the same buffer, were applied overnight at 4°C. After incubation, samples were washed three times for 15 min each in 0.1 % Triton X-100/PEM. Secondary antibodies, diluted in 3 % BSA/0.1 % Triton X-100/PEM, were applied for 2 h at room temperature, followed by sequential washes in 0.1 % Triton X-100/PEM for 5, 10, and 15 min. Samples were mounted in ProLong Glass (Thermo Fisher) or the VECTASHIELD mounting medium.

Oocytes were washed in 0.2 % NGS/PEM for 2 min three times, then fixed in 2 % PFA/0.05 M sorbitol/PEM for 1 h. After fixation, samples were washed again in 0.2 % NGS/PEM for 2 min three times, followed by permeabilization in 0.25 % Triton X-100/PEM for 15 min. Oocytes were then washed three times in drops of 0.1 % NGS/0.01% Triton X-100/PEM and incubated in a fresh drop of the same solution for 10 min three times. Blocking was performed in 2 % NGS/0.1 % Triton X-100/PEM for 1 h. Antibodies were diluted in the same solution and then applied for 2 h at room temperature. Samples were washed sequentially in 0.1 % Triton X-100/PEM for 5, 10, and 15 min at room temperature and mounted in the VECTASHIELD mounting medium.

#### Western blotting

Ovary, brain and small intestine were homogenized through sonication and cell lysates were collected with a 1×sample buffer. Proteins were separated in 5-20 % (w/v) gradient SDS-PAGE precast gels (FUJIFILM Wako) and transferred onto PVDF membranes (Millipore). The membranes were blocked with 3 % skim milk in TBS + 0.1 % Tween 20 (TBST) for 60 min at room temperature and then incubated with primary antibodies overnight at 4°C. After washing with TBST, the membranes were incubated with HRP-conjugated secondary antibodies for 60 min at room temperature. For multiple detections with different antibodies on the same membranes, blotted membranes were soaked in Stripping Solution (FUJIFILM Wako) for 15 min at room temperature and washed sequentially in TBST for 5, 10 and 15 min. Protein bands on the membranes were visualized using an enhanced chemiluminescence (ECL) detection system and imaged with LAS 500 (GE Healthcare). The original uncropped images are shown in Data S1.

#### Generation of reconstituted chimeric follicle

As previously reported,^54^ follicles isolated from WT and *Camsap3*-KO mice were cultured in the two-dimensional system on Petri dishes. For culture medium, pyruvate-free MEMα supplemented with FSH, ITS and 5% FBS was used. On days 2–6 of culture, follicles with diameters of 250–450 μm were chosen, and oocytes and granulosa cells were exchanged to generate chimeric follicles with the following combinations: (1) WT oocyte + WT granulosa cells, (2) WT oocyte + *Camsap3*-KO granulosa cells, (3) *Camsap3*-KO oocyte + WT granulosa cells, and (4) *Camsap3*-KO oocyte + *Camsap3*-KO granulosa cells. To generate chimeric follicles, granulosa cells adhering to the oocyte were removed by gentle pipetting to obtain denuded oocytes, which were then placed onto aggregates of granulosa cells of the indicated genotypes, and the reconstituted chimeric follicles were further incubated. Reconstitution was evaluated based on the adhesion between oocytes and granulosa cells and the presence of a germinal vesicle (GV) within the oocyte on day 9 of culture. Follicles that passed the quality control were applied for observation as reconstituted chimeric follicles.

#### Microscopy

Images were acquired with following microscopes. BZ-X710 All-in-One Fluorescence Microscope (Keyence) equipped with the objective lenses Nikon Plan Apo λ 2×, 10×, 20×, and Nikon CFI Flour 4×. Images were then processed using the BZ X Analyzer software (version 1.3.1.1). LSM980 laser scanning confocal microscope with Airyscan 2 (Zeiss), equipped with the objective lens Zeiss Plan apo 63×. Z-slices were obtained using a 0.15 μm step. Images were processed using the software ZEN (version 3.4.91.00000). Nikon ECLIPSE Ti2 (Nikon) equipped with the objective lenses Nikon plan Flour 10×, 20× and 40× was used for DIC images of oocytes.

#### Image analysis

Fiji (version 1.53),^52^ Imaris (version 10.1.1) and ZEN (version 3.4.91.00000) were used for image analyses. Imaris was used to analyze the TZP length, microtubule thickness, and Camsap3 localization.

#### Follicle count

Sections of the entire ovary, sliced at 5 μm each, were stained with hematoxylin, and microscopic images were acquired. The numbers of primary, secondary, early antral, and Graafian follicles were counted, and the number of follicles per ovary was calculated. Primary follicles were defined as those contain a single layer of GCs, secondary follicles contain two or more layers of GCs with no visible follicular cavities, early antral follicles contain three or more layers of GCs with small follicular cavities, and Graafian follicles contain three or more layers of GCs with a single, united follicular cavity.^31^

The number of primordial follicles was determined as follows: the entire ovary was sliced into 5-μm slices, and every five section was selected for counting. The primordial follicles in the whole section were counted, and the number was multiplied by five, to estimate the number of primordial follicles per ovary.^45^

#### TZP count

Ovarian sections containing each developmental stage of follicles were stained for α-tubulin and F-actin for imaging of TZPs. In the images, a line was drawn above the apical surface of the GCs in parallel. A plot profile of Zen was applied for drawing the line, and the number of peaks of the fluorescence intensities of F-actin and α-tubulin were measured. The ratio of the number of peaks to the total line length was calculated. For classification of TZP types, the software Imaris was used for manually tracing of individual TZPs. Briefly, z-stack images were reconstructed into three-dimensional datasets, and individual TZPs were followed using the ‘ortho slicer’ function, which allows inspection of F-actin and microtubule signals in XY, XZ, YZ planes. These procedures enabled us to discriminate and classify individual TZPs as types 1–3.

#### GDF9 intensity

Secondary and early antral follicles were included in the analyses. Average fluorescence intensities of GDF9 inside an oocyte and in the zona pellucida were both measured. Then, the relative fluorescence intensity of GDF9 was calculated by subtraction of the value in zona pellucida from that in the oocyte.

### Quantification and statistical analysis

#### Statistical analysis

All the experiments were performed at least twice. Data were analyzed using Student’s t-test or one-way ANOVA with Prism software (version 9.4.1 (458); GraphPad). Data are presented as mean ± s.d. The exact n values and the statistical tests used are indicated in the figure legends.

## Supplemental information

Document S1. Figures S1–S4

Data S1. Original uncropped images for western blotting
