# Supplementary material for: Camsap3-mediated microtubules maintain transzonal projections essential for soma–germ communication during ovarian follicle maturation in mice
Source: iScience. 2026 Apr 28;29(6):115911. doi: 10.1016/j.isci.2026.115911 (PMC13200047; doi:10.1016/j.isci.2026.115911)
Supplement: Document S1. Figures S1–S4 [file mmc1.pdf]

iScience, Volume *29*

Supplemental information

Camsap3-mediated microtubules maintain transzonal projections essential for soma–germ communication during ovarian follicle maturation in mice

Akihiro Aikawa, Takao Tsurumaki, Erina Kuranaga, Junya Ito, Mika Toya, and Masamitsu Sato

Figure S1

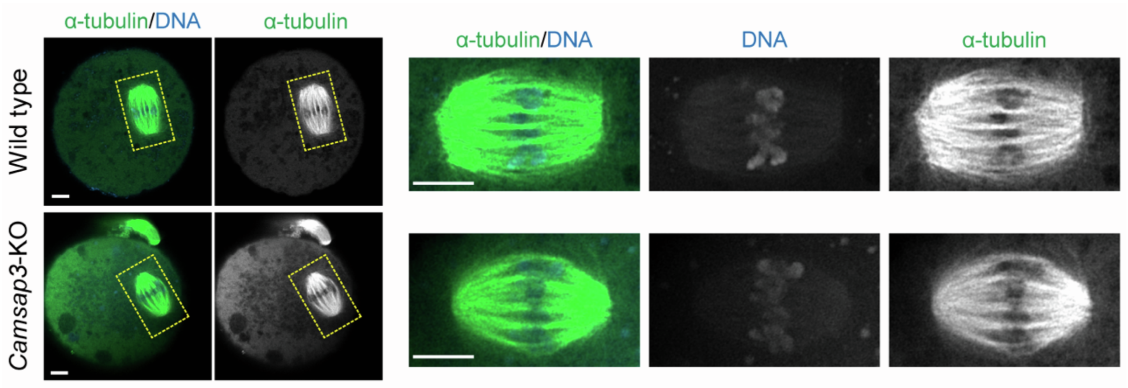

Figure S1. Meiotic spindle assembly in *Camsap3*-KO oocytes is comparable to that observed in WT, Related to Figure 1

Oocytes obtained after superovulation were stained for α-tubulin and DNA. Although oocytes were rarely recovered from *Camsap3*-KO mice, the obtained oocytes appeared to form meiotic II spindles comparable to those in WT oocytes. Insets are shown magnified to the right. Scale bars, 5 μm.

Figure S2

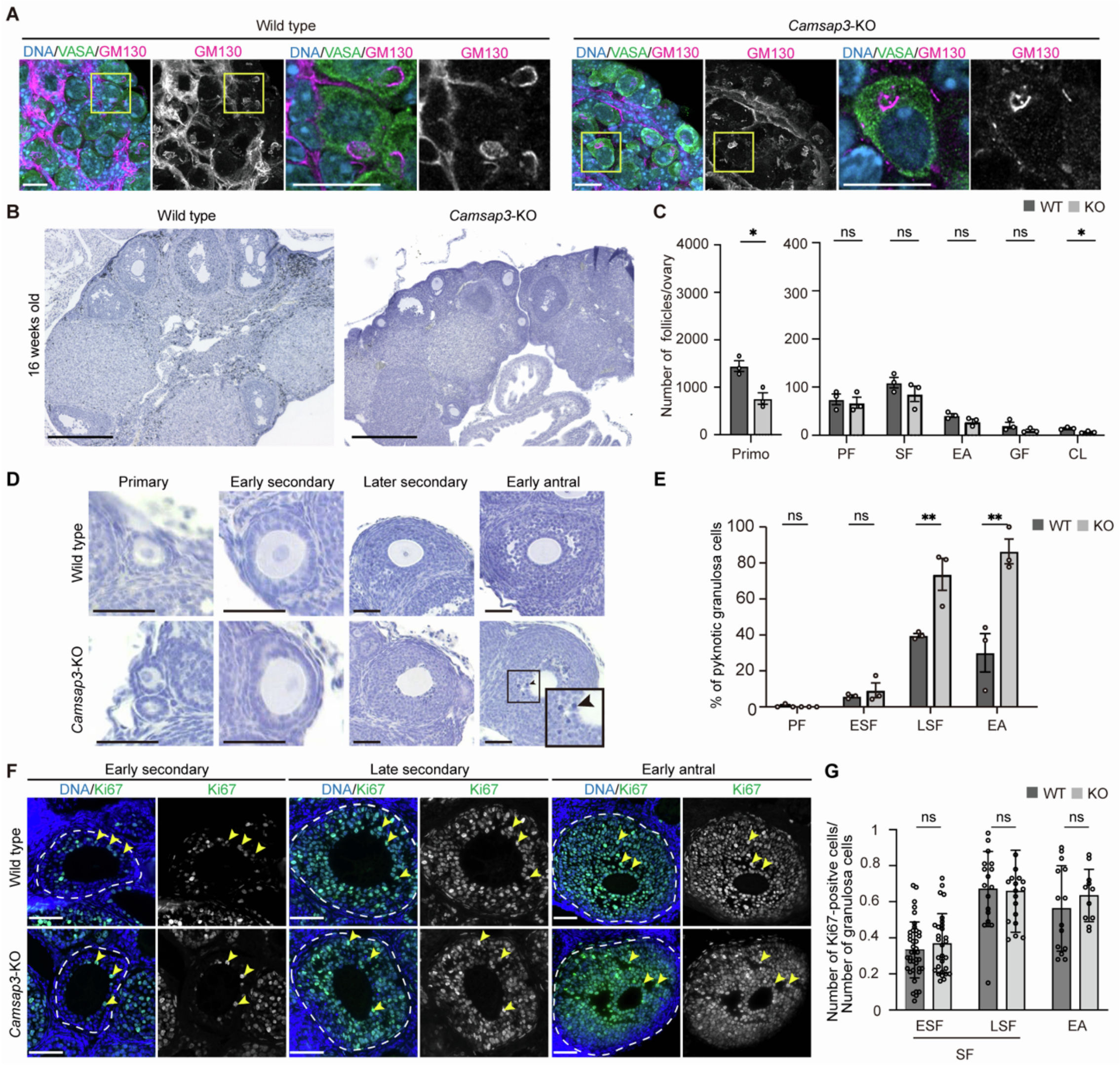

Figure S2. Comparison of follicular development in WT and *Camsap3*-KO ovaries, Related to Figure 2

(A) Representative images of densely packed primordial follicles from WT and *Camsap3*-KO mice. GM130 staining revealed a ring structure in oocytes of both WT and *Camsap3*-KO mice. Insets are magnified and shown on the right. Scale bar, 10 μm.

(B) Ovarian sections from 16-week-old WT and *Camsap3*-KO mice stained with hematoxylin. Scale bar, 300 μm.

(C) Average number of follicles at each stage per ovary: primordial (Primo), primary (PF), secondary (SF), early antral (EA), Graafian (GF) follicles and corpus luteum (CL) (16 weeks: WT, n=3; KO, n=3). *Camsap3*-KO mice had significantly fewer corpora lutea. Bars, mean; error bars, s.d.. *p < 0.05, two-tailed unpaired Student's t-test.

(D) Ovarian sections stained with hematoxylin showing pyknotic GCs (right inset). Scale bar, 50 μm.

(E) Proportion of follicles containing GCs with at least one pyknotic nucleus. (WT, n=3; KO, n=3) In late secondary and early antral follicles, *Camsap3*-KO mice showed an increased percentage of follicles undergoing regression. Bars, mean; error bars, s.d. **$p < 0.01$, two-tailed unpaired Student's t-test.

(F) Immunostaining for Ki67 and DNA in ovarian sections from WT and *Camsap3*-KO mice. Arrowheads indicate Ki67-positive GCs. Scale bar, 50 μm

(G) Percentages of Ki67-positive GCs in a single follicle at the early secondary, late secondary and early antral stages. Numbers of follicles examined: ESF (WT, n=42 follicles; KO, n=32 follicles), LSF (WT, n=17 follicles; KO, n=18 follicles), EA (WT, n=14 follicles; KO, n=8 follicles). Bars, mean; error bars, s.d. two-tailed unpaired Student's t-test.

Figure S3

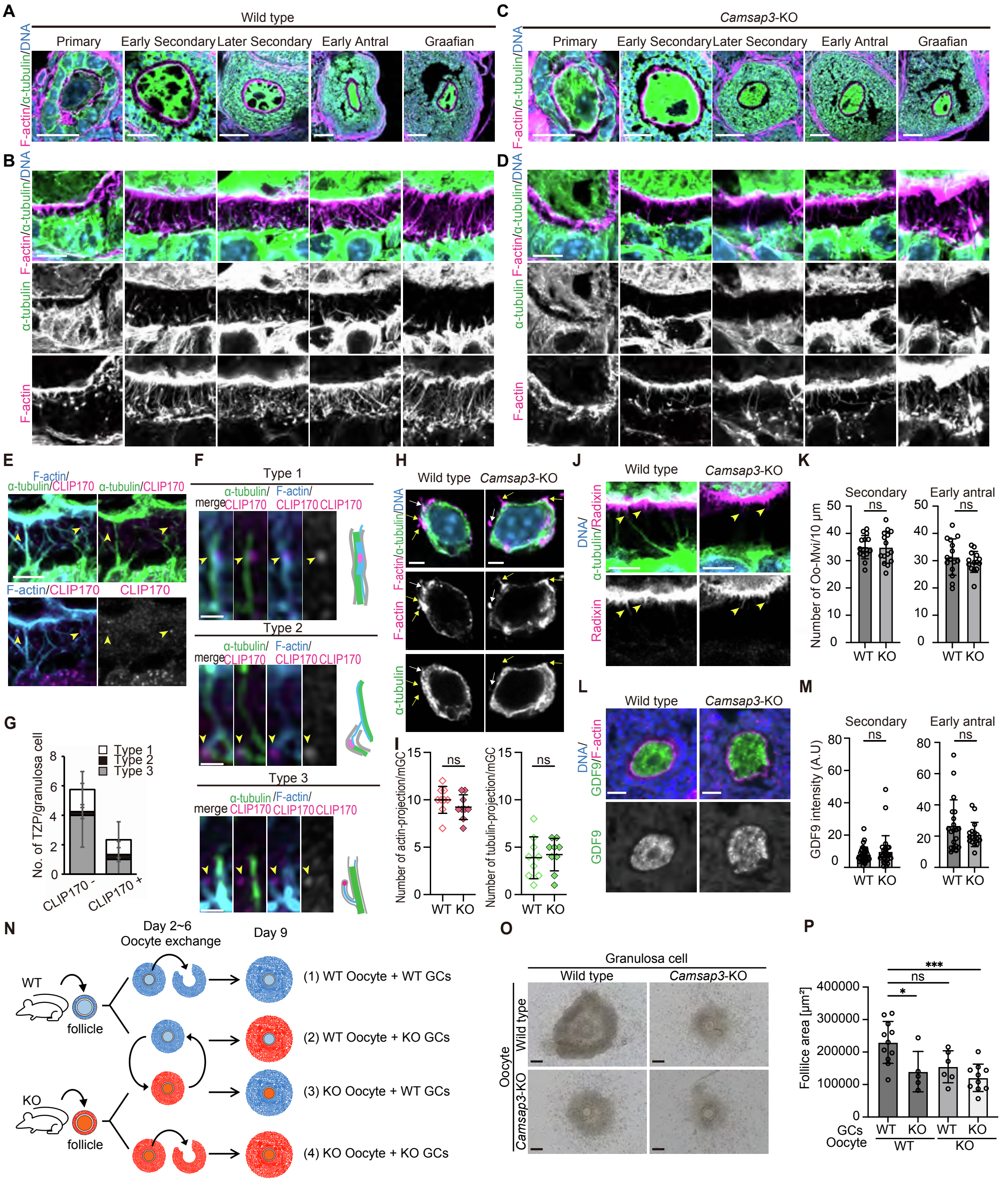

Figure S3. Tubulin- and actin-TZPs in the GC–oocyte communication during follicular development, Related to Figure 3

(A, C) Representative images of follicles at each developmental stage. WT and *Camsap3*-KO ovarian sections were immunostained for F-actin, α-tubulin and DNA. Scale bar, 25 μm (primary, early secondary) 50 μm (later Secondary – Graafian).

(B, D) Magnified images of follicles at the primary, secondary, late secondary, early antral,

and Graafian stages. Scale bar, 5 μm.
(E) Immunostaining for CLIP-170, F-actin and α-tubulin in ovarian sections from WT mice. CLIP-170 localized not only to microtubule tips but also to F-actin. Arrowheads indicate CLIP-170 localization to both microtubule and F-actin. Scale bar, 5 μm.
(F) CLIP-170 localization to TZPs according to the TZP-type defined in Figure 3. Scale bar, 0.5 μm.
(G) The mean number of TZPs per GC with or without CLIP-170 localization. TZPs with CLIP-170 localization were classified by type. Error bars, s.d.
(H) Representative images of mural granulosa cells (mGCs) located in the antral cavity of early antral follicles. For visualization of cellular projections (arrows) from mGCs, ovarian sections from WT and *Camsap3*-KO mice were stained for F-actin, α-tubulin, and DNA. Scale bar, 1 μm. The potential discrepancy in the size of the projections compared to previous work [S1] may be attributed to differing methodologies employed for fixation and visualization.
(I) Number of cellular projections per mGC (WT, n=9 cells; KO, n=9 cells). Data are represented as mean ± s.d. two-tailed unpaired Student's t-test.
(J) Immunostaining for radixin, α-tubulin and DNA in ovarian sections from WT and *Camsap3*-KO mice. Secondary and early antral follicles showed oocyte microvilli (Oo-Mvi, arrowheads), which are rooted in the oocyte. In *Camsap3*-KO follicles, TZP numbers were reduced, whereas oocyte microvilli remained. Scale bar, 5 μm.
(K) The average number of Oo-Mvi per 10 μm at secondary and early antral follicle stages. Numbers of oocytes examined: SF (WT, n=15 cells; KO, n=15 cells), EA (WT, n=15 cells; KO, n=14 cells). Bars, mean; error bars, s.d. two-tailed unpaired Student's t-test.
(L) Immunostaining for GDF9, F-actin and DNA in ovarian sections from WT and *Camsap3*-KO mice. GDF9 localized to the cytoplasm of the oocyte within the secondary and early antral follicles. Scale bar, 20 μm.
(M) The signal intensity of GDF9 in oocytes at the secondary and early antral stages. Numbers of oocytes examined: SF (WT, n=51 cells; KO, n=66 cells), EA (WT, n=33 cells; KO, n=27 cells). Bars, mean; error bars, s.d. two-tailed unpaired Student's t-test.
(N) A schematic for reconstitution assays of chimeric follicles. Follicles were isolated from WT and *Camsap3*-KO mice and cultured *in vitro*. On days 2–6 of culture, follicles with diameters of 250–450 μm were chosen, and oocytes and granulosa cells were exchanged to generate chimeric follicles in the indicated combinations (1)–(4).
(O) Representative images of reconstituted follicles of indicated combinations on day 9 of culture. Scale bar, 100 μm.

(P) The mean area of reconstituted follicles on day 9 (WT oocyte + WT GCs, n=11 follicles; WT oocyte + KO GCs, n=5 follicles; KO oocyte + WT GCs, n=6 follicles; KO oocyte + KO GCs, n=10 follicles). Bars, mean; error bars, s.d. *$p < 0.05$, ***$p < 0.001$, one-way ANOVA.

Figure S4

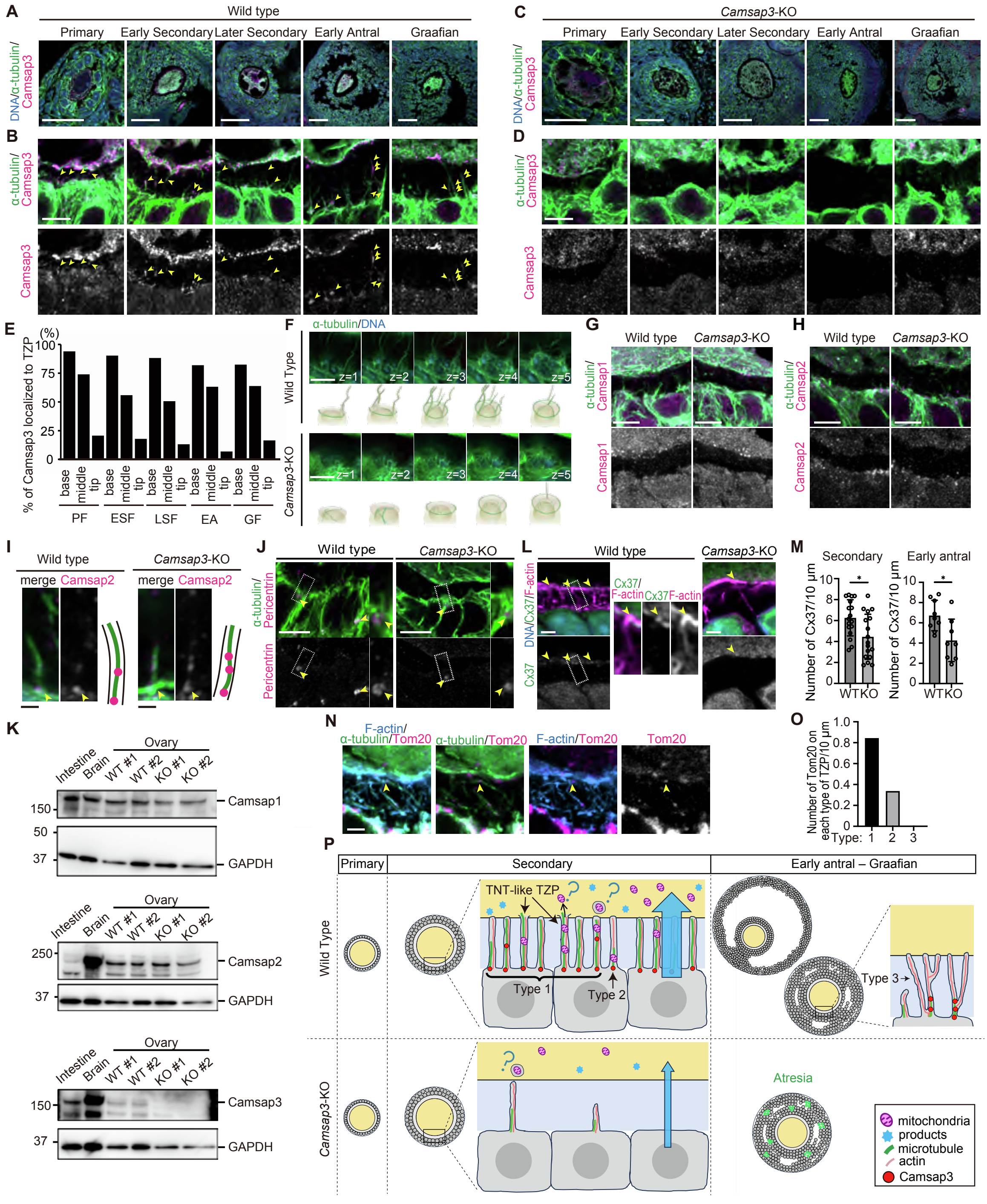

Figure S4. Roles of Camsap3 in the assembly of TZP during follicular development, Related to Figure 4

(A, C) Immunostaining for Camsap3 and α-tubulin showing Camsap3 localization to the apical surface of GCs in secondary and follicular follicles. Scale bars, 25 μm (primary); 50

μm (early secondary – Graafian).

(B, D) Magnified images of the apical surface of GCs showing Camsap3 at the base and along TZPs (yellow arrowheads). Immunostaining with the antibody for Camsap3 [S2] reportedly shows non-specific signals as detected in *Camsap3*-KO (D), and Camsap3 localisation is based on staining that is associated with microtubules within TZPs in WT follicles, a pattern that is absent in KO follicles. Scale bar, 5 μm.

(E) Quantification of the three Camsap3 localization patterns during follicle development.

(F) Apical views of GCs from isolated follicles. In WT, TZPs extended from the cell periphery towards the oocyte; The number of TZP was reduced in *Camsap3*-KO mice. Scale bar, 5 μm.

(G) No detectable Camsap1 was observed in GCs by immunostaining for Camsap1 and α-tubulin. Scale bar, 5 μm.

(H, I) Camsap2 localization to GCs and TZPs indicated by immunostaining for Camsap2 and α-tubulin. Scale bars, 5 μm (H) and 0.5 μm (I).

(J) Representative images showing centrosomal microtubules encapsulated in TZPs. Arrowheads, localization of pericentrin. Scale bar, 5 μm.

(K) Western blot analyses for CAMSAP family proteins in WT and *Camsap3*-KO ovaries, showing no compensatory upregulation of Camsap1 and Camsap2 occurred in the absence of Camsap3. Proteins were prepared from two ovaries from indicated genotypes.

(L) Immunostaining for connexin 37 (Cx37, arrowheads), F-actin and DNA. The tips of TZPs terminated on the oocyte surface via gap junctions (WT). The inset is magnified and shown on the right. Scale bar, 2 μm.

(M) The number of Cx37 signals per 10-μm length along the sectioned oocyte surface in secondary and early antral follicles from WT and *Camsap3*-KO mice. Numbers of follicles examined: SF (WT, n=17 follicles; KO, n=17 follicles), EA (WT, n=8 follicles; KO, n=8 follicles). Bars, mean; error bars, s.d. *$p < 0.05$, two-tailed unpaired Student's t-test.

(N) Immunostaining for Tom20, F-actin and α-tubulin in the WT follicle. Arrowheads indicate Tom20 signals indicating mitochondria detected along TZP microtubules. Scale bar, 2 μm.

(O) The number of Tom20 signals per 10-μm length along the apical surface of GCs, located on each type of TZP (Type 1–3) in WT secondary follicles. Tom20 signals were not detected along the observed Type 3-TZPs, as TZPs in this category were not frequently observed in seconary follicles.

(P) A model for Camsap3 function in ovarian follicles. Microtubules contribute to the maintenance of TZPs, which is essential for follicle development. Camsap3-dependent microtubules promote the temporal transition of TZP morphology during folliculogenesis. In

the early stages, the majority of TZPs are classified as type 1; some penetrate the oocyte (TNT-like TZPs), whereas others establish contact with the oocyte surface via gap junctions. Although both types of TZPs may mediate the transfer of macromolecules and organelles, such as mitochondria, TNT-like TZPs are likely to do so more efficiently, as they deliver cargo directly into the oocyte cytoplasm, whereas other TZPs rely on vesicular transport mechanisms. In later stages, more than 50% of TZPs transform into branched structures (Type 3). In *Camsap3*-KO follicles, the number of TZPs is significantly reduced during the early stages, which may impair cargo transport into the oocyte. At later stages, knockout follicles fail to develop fully and consequently undergo atresia.

REFERENCES

S1. Baena, V., and Terasaki, M. (2019). Three-dimensional organization of transzonal projections and other cytoplasmic extensions in the mouse ovarian follicle. Sci Rep-uk *9*, 1262. https://doi.org/10.1038/s41598-018-37766-2.

S2. Tanaka, N., Meng, W., Nagae, S., and Takeichi, M. (2012). Nezha/CAMSAP3 and CAMSAP2 cooperate in epithelial-specific organization of noncentrosomal microtubules. P Natl Acad Sci Usa *109*, 20029–20034. https://doi.org/10.1073/pnas.1218017109.
