# Supplementary figures and images for: Camsap3-mediated microtubules maintain transzonal projections essential for soma–germ communication during ovarian follicle maturation in mice
Source: iScience. 2026 Apr 28;29(6):115911. doi: 10.1016/j.isci.2026.115911 (PMC13200047; doi:10.1016/j.isci.2026.115911)

Camsap1  
for Figure S4K upper

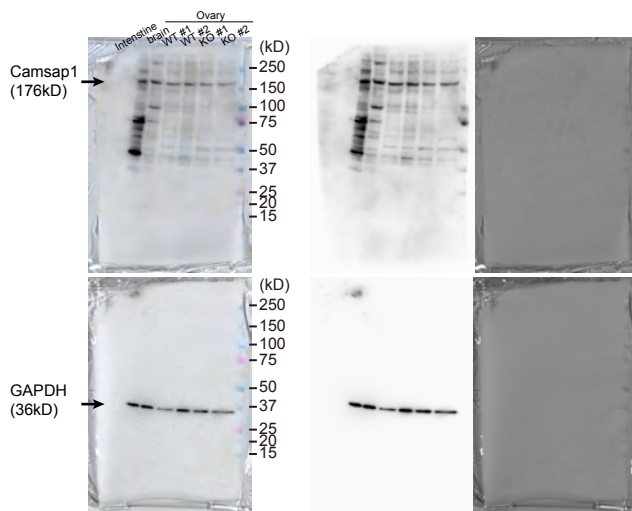

Camsap2  
for Figure S4K middle

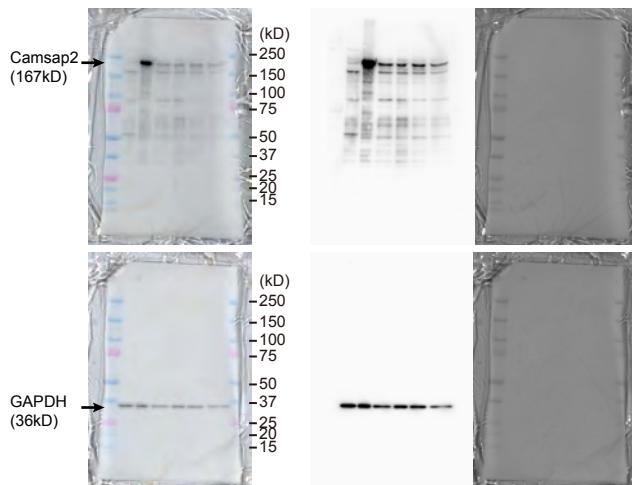

Camsap3  
for Figure S4K lower

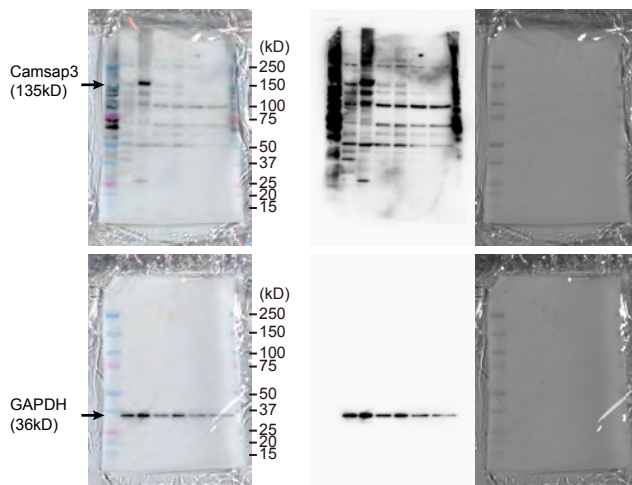

Supplement: Data S1. Original uncropped images for western blotting [file mmc2.pdf]
